# Overexpression of Bruton Tyrosine Kinase Inhibits the Proliferation, Migration, and Invasion of Non-Small Cell Lung Cancer Cells

**DOI:** 10.1155/2023/3377316

**Published:** 2023-08-18

**Authors:** Wenjia Ren, Cheng Yue, Linjun Liu, Licheng Du, Ke Xu, Yubai Zhou

**Affiliations:** ^1^Department of Biotechnology, College of Life Science and Chemistry, Beijing University of Technology, Chaoyang, Beijing, China; ^2^NHC Key Laboratory of biosafety, National Institute for Viral Disease Control and Prevention, Changping, Beijing, China

## Abstract

Lung cancer is one of the most lethal malignant tumors in the world. Non-small cell lung cancer (NSCLC) is the most common pathological subtype. However, the molecular mechanism of NSCLC progress is still unclear. We extracted the expression data of the Bruton's tyrosine kinase (*BTK*) gene in NSCLC tissues from the TCGA database. The results of paired *t*-test showed that the *BTK* gene was significantly underexpressed in NSCLC tissues. To further verify the above results, we detected the expression of the *BTK* gene in NSCLC cell lines A549, H1299, and H1650 at the RNA and protein levels by real-time fluorescent quantitative polymerase chain reaction and Western Blot analysis, respectively. The results showed that BTK was low expressed in NSCLC tissues and cells. More importantly, the expression of the *BTK* gene is also significantly related to the patient's age, gender, tumor range (T), lymph node invasion (N), tumor stage, and prognosis, and its expression level gradually decreases with the progress of the disease. It is speculated that BTK may be an independent prognostic factor of NSCLC. Our experimental results are consistent with the above clinical correlation analysis results. Overexpression of BTK can significantly inhibit the proliferation, migration, and invasion of NSCLC cells and can block the G0/G1 tumor cell cycle, indicating that overexpression of BTK can inhibit the growth, migration, and invasion of NSCLC cells.

## 1. Introduction

Lung cancer is a malignant tumor with extremely high incidence rates and mortality rates worldwide, which seriously affects human health [1, 2]. According to statistics, there will be about 2.2 million new cases, and 1.8 million deaths of lung cancer worldwide in 2020, and lung cancer deaths account for 18.0% of all cancer deaths [3]. According to the different pathological characteristics, lung cancer is mainly divided into non-small cell lung cancer (NSCLC) and small-cell lung cancer (SCLC) [4]; NSCLC can be further divided into lung squamous cell carcinoma, lung adenocarcinoma (LUAD) and large cell carcinoma, among which LUAD is the most common, accounting for about 40% of all new cases of lung cancer [5]. Although clinical treatments for lung cancer have made breakthroughs in radiotherapy, chemotherapy, surgical resection, and new immunotherapy and targeted therapies [6–8], the prognosis for patients with NSCLC is still unsatisfactory [9], and the 5-year overall survival rate for patients still does not exceed 20% [3]. Therefore, further research on the molecular mechanism of lung cancer development, the search for new drug targets, and the development of personalized treatment plans, including targeted and immunotherapy for different patients with NSCLC, will be the key to lung cancer treatment in the future.

Bruton's tyrosine kinase (BTK) is a nonreceptor tyrosine kinase, and in the human genome, the BTK gene is located in the Xq21.322.1 region [10]. BTK is a member of the Tec protein-tyrosine kinase (TEC) kinase family [11]. TEC family kinases are preferentially expressed in the hematopoietic system [12]. Members of this family play an important role in innate and adaptive immune responses, and they can assist T-cell differentiation [11, 12]. BTK protein was initially concerned as a drug target for B-lymphocyte malignancies, and it can promote the occurrence of B lymphoblastic lymphoma [13, 12]. A key feature of BTK is that it interacts with the PI3K/AKT signaling pathway and functions as an upstream of nuclear factor kappa B and extracellular signal-regulated kinase, thereby affecting the proliferation, survival, and differentiation of lymphoma cells [14]. Subsequent studies found that BTK was detected in various cancers, including colon cancer and breast cancer [15–17], suggesting that BTK protein may play a role in the development of other cancers. At present, a variety of BTK inhibitors, such as ibrutinib, have been used in clinical-targeted therapy and achieved good therapeutic results [18–21]. BTK/ITK dual inhibitors have shown some therapeutic potential in regulating immune response and COVID-19 [22]. In lung cancer, the BTK inhibitor ibrutinib effectively inhibits the proliferation of some epidermal growth factor receptor (EGFR) mutant lung cancer cells by inhibiting the autophosphorylation of EGFR [23]. Recent studies have found that p65BTK is a new BTK subtype in Kirsten rat sarcoma virus oncogene-activated colon cancer and NSCLC [24]. The p65BTK is overexpressed in NSCLC and maybe a new drug target [25]. At the same time, studies have also found that the inhibitor ibrutinib seems to be a new therapeutic candidate for NSCLC patients with EGFR mutation. The contradiction of the clinical effects of BTK inhibitors in tumor therapy proves that BTK plays a heterogeneous and complex role in the development of different types of tumors. However, the biological role of BTK in the development of lung cancer and its role in NSCLC has not been studied. By analyzing the expression of BTK in NSCLC, the effects of BTK on the proliferation, migration, invasion, and apoptosis cycle of NSCLC cells were investigated.

## 2. Methods and Materials

### 2.1. Gene Expression Analysis Based on TCGA Database

To explore whether the expression of *BTK* gene in cancer and normal tissues is different, we first extracted FPKM format expression data of the *BTK* gene in LUAD and paired adjacent tissues from the TCGA database (https://portal.gdc.cancer.gov/) in 2019 and then converted FPKM format expression data to TPM (transcripts per kilobase of exit model per million mapped reads) format and log_2_ (TPM + 1) data conversion. Finally, we analyzed the expression difference by paired *t*-test. Accordingly, we also extracted the expression values of two prognostic genes in LUAD and normal tissues and analyzed the difference of expression by Wilcoxon rank sum test after data conversion according to the above steps.

### 2.2. Correlation between Prognostic Genes and Clinicopathological Characteristics of LUAD Patients

To analyze whether the expression of prognostic gene BTK is related to the course of LUAD patients, we dichotomized the clinicopathological indicators of the samples in the LUAD cohort, such as sex, age, T, N, M, and tumor stage, and analyzed whether the expression level of the above prognostic genes is different between the dichotomies of different clinicopathological indicators by Wilcoxon rank sum test.

### 2.3. Independent Prognostic Factor Analysis

We analyzed whether BTK can be used as an independent prognostic factor of LUAD by univariate and multivariate Cox regression and referring to other commonly used clinicopathological indicators.

### 2.4. Cell Culture

The human lung cancer cell lines H1650, H1299, H460, and A549 were purchased from the National Collection of Authenticated Cell Cultures (Shanghai, China). The normal human bronchial epithelial cell lines 16HBE are obtained from the Chinese Academy of Cell Resource Center (Shanghai, China). H1650, H1299, H460, and 16HBE were cultured in Roswell Park Memorial Institute 1640 medium (Thermo Fisher Scientific, USA) supplemented with 10% fetal calf serum (fetal bovine serum (FBS), Thermo Fisher Scientific, USA). A549 were cultured in Dulbecco's modified Eagle's medium (DMEM, Thermo Fisher Scientific, USA) supplemented with 10% fetal calf serum (FBS, Thermo Fisher Scientific, USA). All cells were maintained in a 37°C, 5% CO_2_ incubator (Thermo Fisher Scientific, USA).

### 2.5. RNA Extraction and Quantitative RT-PCR

By the manufacturer's instructions, the total RNA was extracted from culture cells or tissues using the Trizol Reagent (Ambion, USA). The first-strand cDNA was synthesized by the Goldenstar™ RT6 cDNA Synthesis Kit Ver 2 (Beijing TsingKe Biotech Co., Ltd., China). Real-time fluorescent quantitative polymerase chain reaction (RT-qPCR) was performed using the SYBR Premix Ex Taq kit (Takara Biotechnology Co., Ltd., China) on a ViiA7 real-time PCR (Applied Biosystems, Foster City, CA, USA). The reaction conditions are as follows: predenaturation 95°C to 15 s; 95°C to 15 s, 55°C 30 s, 72°C 45 s; melting curve. The whole qPCR reaction lasted 40 cycles. All RT-qPCR primer sequences were designed and synthesized by Beijing TsingKe Biotech Co., Ltd. (Beijing, China). The primers used in this study were the following: BTK, forward 5′-GTCAGAGACTCCAGCAAAGCTG-3′ and reverse 5′-TACTGGCTCTGAGGTGTGGAAC-3′, GAPDH, forward 5′-GTCTCCTCTGACTTCAACAGCG-3′ and reverse 5′-ACCACCCTGTTGCTGTAGCCAA-3′.

### 2.6. Cell Transfection

Use the jetPRIME® transfection reagent (Polyplus, France) for cell transfection according to the manufacturer's instructions. Empty plasmid (pcDNA3.1-HA, ZT119) were purchased from Fenghui Biological Company (Hunan, China). BTK overexpression plasmid (pcDNA3.1-HA-BTK) was constructed by Fenghui Biological Company (Hunan, China).

### 2.7. Western Blot

Total protein was extracted from cell lysates after cells were harvested and centrifuged at 4°C and 16,000 × *g* for 20 min. Protein concentrations were measured by the BCA method (Beijing Dingguo Changsheng Biotechnology Co., Ltd. company, China). Subsequently, proteins were separated via 12% sodium dodecyl sulfate–polyacrylamide gel electrophoresis for 1.5 hr and transferred onto polyvinylidene fluoride (PVDF) membranes using a wet transfer electrophoresis tank for 1 hr. Sealed with 5% skim milk for 1 hr at room temperature. Wash with Tris-buffered saline with Tween-20 (TBST) solution three times for 10 min each time. PVDF membranes were then incubated overnight at 4°C with the following primary antibodies: anti-BTK (1 : 1,000; cat. no. #8547S; Cell Signaling Technology, USA); anti-glyceraldehyde-3-phosphate dehydrogenase (GAPDH) (1 : 1,000; cat. no. ab181602; Abcam, UK). After being washed with TBST twice, the membranes were further incubated with horseradish peroxidase-conjugated secondary antibody (1 : 2,000; cat.no. ab6721; Abcam, UK). The visualization of bands was performed with a hypersensitive luminescence development kit (Beyotime Biotechnology, Shanghai, China) in Tanon 5200 chemiluminescence imaging system (Tanon, Shanghai, China).

### 2.8. Cell Count Kit 8 (CCK-8) Assay

The changes in cell viability were measured using the CCK8 assay. After 24 hr of transfection, the concentration was set to per well 5 × 10^3^ cells were inoculated into 96 well plates and divided into four groups: 0, 24, 48, and 72 hr. Detect the optical density value at each of the four time points mentioned above, add 10 *µ*L CCK8 (Beyotime Biotechnology, Shanghai, China) solution was added into each well to incubate the cells, and the absorbance at 450 nm was assessed by a microplate auto-reader (Perkin Elmer, USA). The assay was in triplicate and repeated at least three times.

### 2.9. Wound Healing

Cells were inoculated into the 6-well plates for transfection, and a 10 *μ*L pipette tip was used to create a wound on the monolayer of transfected cells. Fresh 1% FBS medium was placed into the plates immediately to discard the floating cells, and the scratch, as well as surrounding cells, were recorded. Then, photographs were obtained at 24 hr to determine the wound closure using a microscope (Olympus, Tokyo, Japan). Calculate the cell confluence rate. The assay was in triplicate and repeated at least three times.

### 2.10. Transwell Invades

This assay was performed using the transwell chamber with a pore size of 8 *μ*m and 24-well plates. Briefly, the transwell chamber separated the plate into upper and lower chambers. The upper chamber was seeded with 1 × 10^5^ cells in a medium without FBS, while the lower chamber was supplemented with a medium containing 20% FBS. After 48 hr, cells were fixed in 4% paraformaldehyde and stained with crystal violet. Invasive cells were counted and visualized by an inverted microscope (Olympus, Tokyo, Japan). The assay was in triplicate and repeated at least three times.

### 2.11. The Cell Cycle Was Detected by Flow Cytometry

After transfection of A549, H1299, and H1650 cells for 24 hr, the cells were resuspended with 1 mL of precooled 70% ethanol and placed at 4°C overnight. After removing ethanol, phosphate-buffered saline (PBS) (Thermo Fisher Scientific, USA) containing RNase A (Yeasen Biotechnology Co., Ltd., Shanghai, China) was added to the sample and placed at room temperature for 30 min. Centrifuge to remove the supernatant and take 200 *μ*L PBS resuspends the cells and transfers them to a clean flow tube after filtering through a 300 mesh nylon net. Add 5 *μ*L PI (Yeasen Biotechnology Co., Ltd., Shanghai, China) to each sample was incubated in the dark for 30 min and detected by flow cytometry (BD Biosciences, USA) within 1 hr. The assay was in triplicate and repeated at least three times.

### 2.12. Statistical Analysis

Statistical analyses were completed using GraphPad Prism 9 software. Normally distributed variables were analyzed using Student's *t*-test, while nonnormally distributed variables were analyzed using Kruskal–Wallis. *P* ≤ 0.05 was set as the threshold for statistical significance.

## 3. Results

### 3.1. Expression Analysis of Prognostic Gene in LUAD Samples and Cells

**Figure 1:**
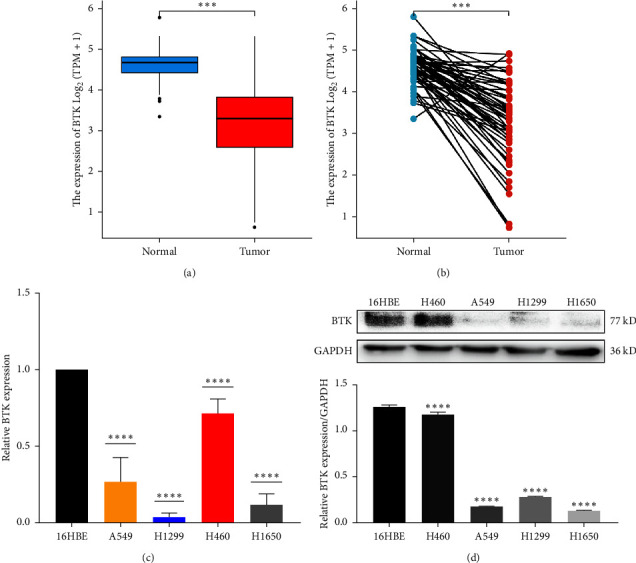
Low expression of BTK in non-small cell lung cancer. (a) Differential analysis of BTK gene expression in lung adenocarcinoma and normal tissues; (b) differential analysis of BTK gene expression in lung adenocarcinoma and paired paraneoplastic tissues. (c) Quantitative RT-PCR analysis of BTK expression levels in normal bronchial epithelial cell 16HBE and non-small cell lung cancer cell line; (d) western blot analysis of BTK expression levels in normal bronchial epithelial cell 16HBE and non-small cell lung cancer cell lines. The results are expressed as the average ± SDs of three independent experiments, with each experiment repeated three times.  ^*∗∗∗∗*^*P* < 0.0001.

TCGA expression profile data analysis showed that BTK showed low expression in LUAD tissues compared with normal and paraneoplastic tissues (Figures 1(a) and 1(b)). To verify the above results, RT-qPCR and Western Blot were used to detect RNA and protein levels, respectively. The results showed that the expression level of BTK in LUAD cells was significantly lower than that in control cells (16HBE), which was consistent with the results of bioinformatics analysis (Figures 1(c) and 1(d)) ( ^*∗∗∗∗*^*P* < 0.0001).

### 3.2. Correlation between BTK and Clinicopathological Features and Independent Prognostic Factors

**Figure 2:**
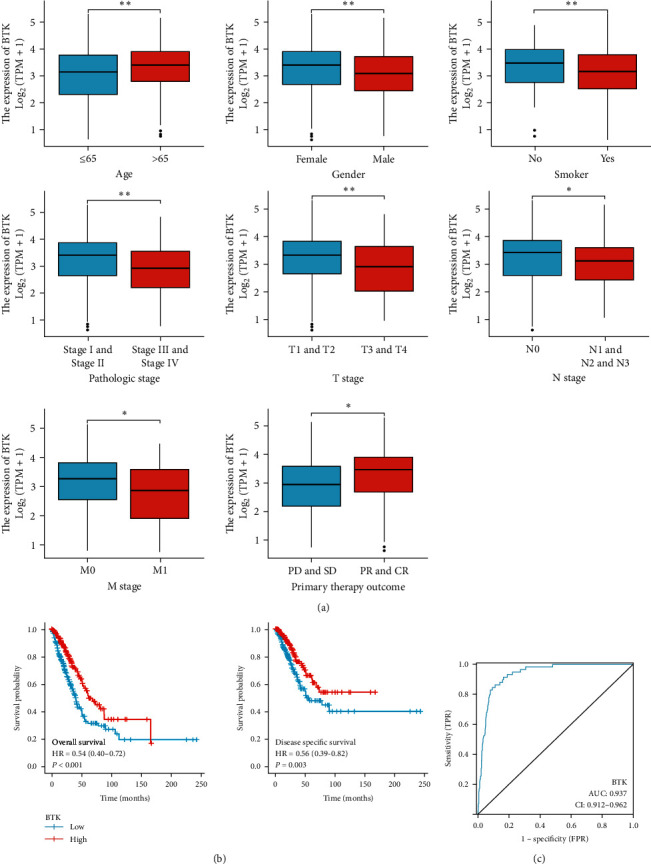
There is a significant correlation between multiple clinical and pathological features of BTK and LUAD patients. (a) In Wilcoxon rank sum test analysis, the correlation between BTK and patient age, sex, smoking, pathological stage, T, N, M stage, and main treatment effect; (b) in the K–M survival analysis, the association between BTK and patients' OS and DSS; (c) in receiver operating characteristic curve, the accuracy of prediction is as high as 0.937.  ^*∗*^*P* < 0.05,  ^*∗∗*^*P* < 0.01.

To further explore the correlation between BTK and the clinicopathological characteristics of LUAD patients, we analyzed whether there were differences in the expression level of the *BTK* gene between the patient's age, sex, smoking, pathological stage, and T, N, M stage, and the main therapeutic effects by Wilcoxon rank sum test. The results showed that there were significant differences in the expression of the *BTK* gene between patients' age, sex, smoking, pathological stage, T, N, M stage, and initial treatment effect ( ^*∗*^*P* < 0.05), and the expression level of BTK decreased with the development of LUAD (Figure 2(a)). K–M survival analysis results showed that patients with LUAD with high expression of prognostic genes BTK had a better prognosis (Figure 2(b)). The receiver operating characteristic curve shows that the accuracy of our prediction is as high as 0.937 (Figure 2(c)). To explore whether the prognostic gene BTK can be used as an independent prognostic factor of LUAD, a univariate and multivariate Cox regression analysis was conducted on 526 lung cancer patients with complete clinical information. The results showed that in univariate analysis, T, N, M, tumor stage, and the expression level of BTK were significantly correlated with the prognosis of patients ( ^*∗*^*P* < 0.05), while in multivariate analysis, only N and BTK were significantly correlated with the prognosis of patients ( ^*∗*^*P* < 0.05). This indicates that the *BTK* gene can be used as an independent prognostic factor of LUAD (Table 1).

### 3.3. BTK Inhibits Cell Proliferation (CCK-8)

**Figure 3:**
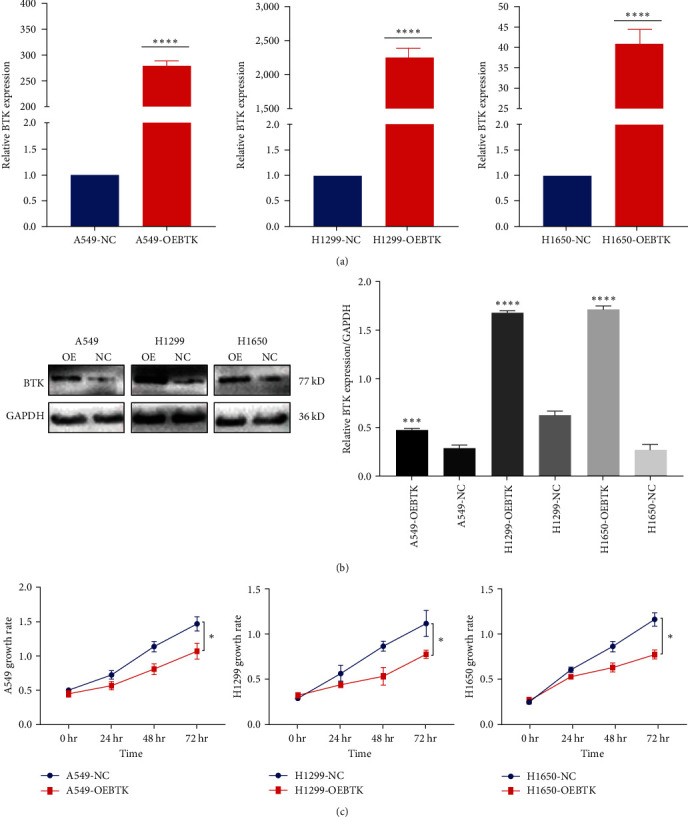
Overexpression of BTK inhibits the proliferation of A549, H1299, and H1650 cells. (a) After transfecting A549, H1299, and H1650 cells with BTK overexpression plasmids or control plasmids, the expression of BTK was analyzed using RT-qPCR. (b) Western blot was used to detect the overexpression efficiency of BTK in cancer cell lines; (c) CCK8 experiment detected the effect of overexpression of BTK on the proliferation of A549, H1299, and H1650 cells. The above results are represented as the average ± SD of three independent experiments, each in triplicate.  ^*∗*^*P* < 0.05,  ^*∗∗∗*^*P* < 0.001,  ^*∗∗∗∗*^*P* < 0.0001.

Its overexpression vector was designed to study the function of BTK in lung cancer cell lines. To verify whether the BTK overexpression vector can effectively express in target cells, we transfected the overexpression vector carrying the *BTK* gene and empty vector into LUAD cells, respectively, and verified their expression by RT-qPCR and Western Blot experiments. The results of relevant validation confirmed that the mRNA level and protein expression of the *BTK* gene in the cells of the experimental group transferred to cells containing prognosis gene plasmid increased significantly compared with the control group (Figures 3(a) and 3(b)) ( ^*∗*^*P* < 0.05,  ^*∗∗*^*P* < 0.01). A549, H1299, and H1650 cell lines were selected to verify the biological function of BTK because of the low level of BTK expression in these cells. BTK overexpression vector or empty vector was transfected into three lung cancer cell lines. CCK-8 assay showed that compared with the vector NC group, the proliferation of the overexpression vector group cell lines was significantly inhibited 24 hr later (Figure 3(c)).

### 3.4. BTK Inhibits Migration and Invasion of Lung Cancer Cells

**Figure 4:**
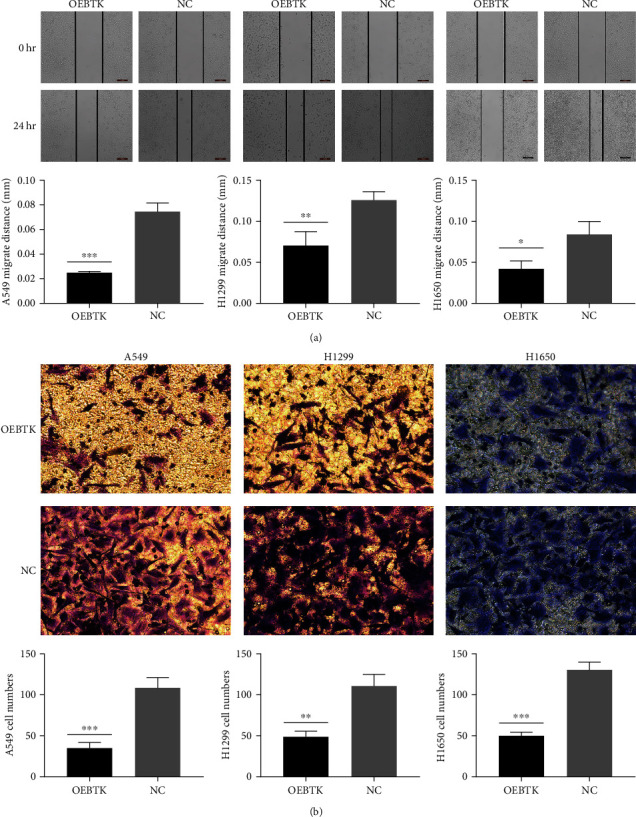
Overexpression of BTK inhibits the migration and invasion ability of A549, H1299, and H1650 cells. (a) The wound healing experiment was used to detect the effect of BTK overexpression on the cell migration ability of A549, H1299, and H1650 cells. (b) The Transwell experiment detected the effect of BTK overexpression on the invasiveness of A549, H1299, and H1650 cells. The above results are represented as the average ± SD of three independent experiments, each in triplicate.  ^*∗*^*P* < 0.05,  ^*∗∗*^*P* < 0.01,  ^*∗∗∗*^*P* < 0.001. The scale bar represents 100 *μ*m.

To explore whether BTK affects the migration and invasion of lung cancer cell lines, we transfected cells with BTK expression vectors to verify the role of BTK in the migration and invasion of lung cancer cell lines. The wound-healing assay demonstrated that the BTK overexpressing cells had weaker migratory ability compared to the empty vector group (Figure 4(a)). Transwell invasion assays showed that overexpression of BTK remarkably suppressed the invasion of A549, H1299, and H1650 cells (Figure 4(b)).

### 3.5. BTK Inhibits Cell Cycle Progression

**Figure 5:**
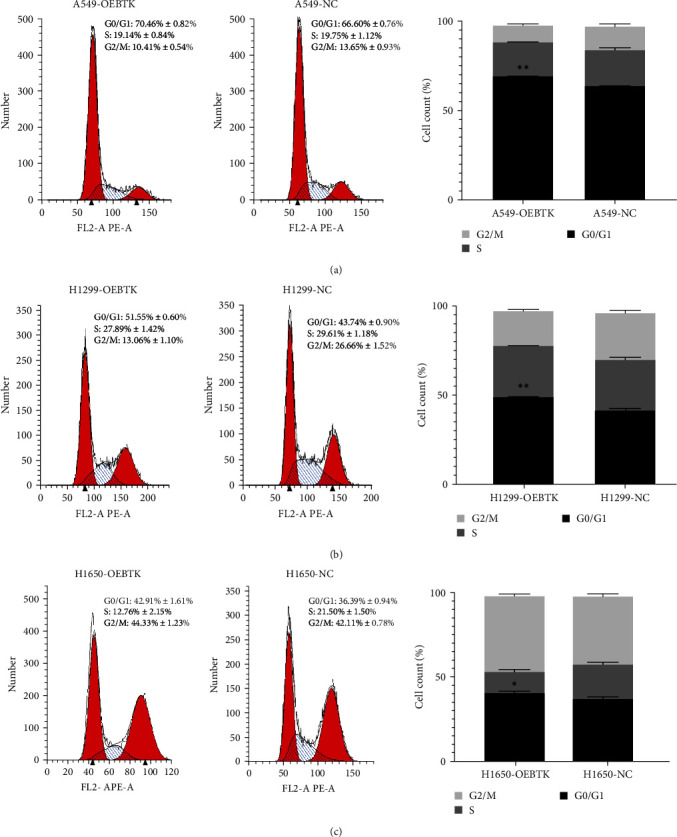
Overexpression of BTK blocks the cell cycle of A549, H1299, and H1650 cells. (a–c) The regulation of overexpression of BTK on A549, H1299, and H1650 cell cycles was detected using flow cytometry. The above results are represented as the average ± SD of three independent experiments, each in triplicate.  ^*∗*^*P* < 0.05,  ^*∗∗*^*P* < 0.01.

Flow cytometry was used to verify the role of prognostic genes in inducing the cell cycle of LUAD. The results showed that the proportion of A549 cells transfected with the *BTK* gene in the G0/G1 phase was 70.46% ± 0.82%, and the proportion of A549 cells transfected with empty vector in the G0/G1 phase was 66.6% ± 0.76%. These results indicate that the *BTK* gene may affect cell activity by blocking G0/G1 LUAD cells. (Figure 5(a)–5(c)).

## 4. Discussion

Lung cancer is one of the most malignant tumors in the world, but the pathogenesis and pathogenesis of lung cancer have not been fully clarified. In our study, we first explored the biological function of BTK in NSCLC. Compared with human normal lung epithelial cell 16HBE, the expression of BTK mRNA and protein in the lung cancer cell line was significantly downregulated. Further research has shown that overexpression of BTK inhibits the proliferation, migration, and invasion of NSCLC cells in vitro. In addition, BTK can block NSCLC cells in G0/G1 phase. These results indicate for the first time that BTK may be a potential tumor suppressor in lung cancer.

BTK located in the cytoplasm, is a non-receptor tyrosine kinase, belonging to the TEC family of kinases [26–28]. Its gene encodes a protein with 659 amino acids, including a single kinase domain and multiple protein interaction domains [29]. BTK regulates the function of B cells in host defense and autoimmunity by participating in the BCR signaling pathway [13, 18]. *BTK* gene mutation leads to human X-linked agammaglobulinemia and mouse X-linked immunodeficiency [13]. Several previous studies have shown that BTK has a certain role in promoting tumor occurrence and development. For example, BTK regulates tissue plasminogen activator-induced invasion of breast cancer cells by activating matrix metalloproteinase-9 [17]. In bone marrow cells, BTK has been proved to be activated by hypoxia-induced mitotic factor receptor, which stimulates the migration of bone marrow cells [30]. BTK is also oncogenic in B-cell malignancies [31], where BTK inhibits Wnt-*β*-Catenin signaling by increasing the abundance of CDC73 protein [32]. BTK is highly expressed in malignant cells and certain cell lines of many patients with multiple myeloma (MM). Chauhan et al. [33] showed that BTK mRNA expression was 2.3-fold increase in 6 MM patient specimens compared to normal human marrow precursors. In chronic lymphocytic leukemia, cell proliferation is blocked by inhibiting BTK-mediated signal transduction [34]. In recent years, some researchers have shown that targeting the BTK signal in the solid tumor microenvironment is feasible for cancer treatment [26, 28]. Now, there are many BTK inhibitors on the market, which have been used in the clinical treatment of blood malignancies and have achieved good therapeutic effects. However, BTK can also perform oncogenic functions in other tumor cells [35]. Bi et al. [36], based on the mining of TCGA data, showed that BTK has the potential to become a prognostic factor of LUAD and an indicator of tumor microenvironment remodeling. Their analysis showed that the expression of BTK was negatively correlated with the clinical pathological characteristics (clinical stage, distant metastasis) of LUAD patients and positively correlated with survival. Our study found that BTK can inhibit proliferation, migration, and invasion of NSCLC cell lines and even act as a cell cycle blocker. Taken together, BTK may have opposite effects on different types of tumors.

In conclusion, our study confirms that BTK can inhibit the proliferation, invasion, and metastasis of NSCLC. Although this study needs further validation in vivo, it preliminarily proves that BTK, as a tumor suppressor factor, plays a role in NSCLC and is a potential biomarker and therapeutic target.

## 5. Conclusions

Therefore, it can be concluded from our experiments that BTK is lowly expressed in NSCLC, and overexpression of BTK suppresses the tumor phenotype of NSCLC cells. It indicates that BTK has potential antitumor effects and may become a potential biomarker and therapeutic target for NSCLC.

## Figures and Tables

**Table 1:** Independent prognostic analysis of BTK and clinicopathological parameters.

Factor	Samples (*N*)	Single factor analysis		Multifactor analysis	
		Hazard ratio (95% CI)	*P* value	Hazard ratio (95% CI)	*P* value
Gender	526				
Female	280				
Male	246	1.070 (0.803–1.426)	0.642		
Age	516				
≤65	255				
>65	261	1.223 (0.916–1.635)	0.172		
T	523				
T1 and T2	457				
T3 and T4	66	2.317 (1.591–3.375)	**<0.001**	1.818 (1.142–2.892)	**0.012**
N	510				
N0	343				
N1 and N2 and N3	167	2.601 (1.944–3.480)	**<0.001**	2.114 (1.440–3.103)	**<0.001**
M	377				
M0	352				
M1	25	2.136 (1.248–3.653)	**0.006**	1.370 (0.720–2.605)	0.338
Cancer stage	518				
I and II stage	411				
III and IV stage	107	2.664 (1.960–3.621)	**<0.001**	1.279 (0.785–2.081)	0.323
BTK	526				
Low-level	262				
High expression	264	0.539 (0.401–0.725)	**<0.001**	0.546 (0.383–0.779)	**<0.001**

Bold values signify the statistically significant (*P* value ≤ 0.05).

## Data Availability

Raw data are available from the corresponding author upon reasonable request.
